# Venom Proteins of the Parasitoid Wasp *Nasonia vitripennis*: Recent Discovery of an Untapped Pharmacopee

**DOI:** 10.3390/toxins2040494

**Published:** 2010-03-30

**Authors:** Ellen L. Danneels, David B. Rivers, Dirk C. de Graaf

**Affiliations:** 1 Laboratory of Zoophysiology, Ghent University, K.L. Ledeganckstraat 35, B-9000 Ghent, Belgium; Email: Dirk.deGraaf@UGent.be; 2 Department of Biology, Loyola University Maryland, 4501 N. Charles St., Baltimore, MD 21210 USA; Email: DRivers@loyola.edu

**Keywords:** *Nasonia*, venom, immunity, phenoloxidase, coagulation, development, lipid, apoptosis, nutrient

## Abstract

Adult females of *Nasonia vitripennis* inject a venomous mixture into its host flies prior to oviposition. Recently, the entire genome of this ectoparasitoid wasp was sequenced, enabling the identification of 79 venom proteins. The next challenge will be to unravel their specific functions, but based on homolog studies, some predictions already can be made. Parasitization has an enormous impact on hosts physiology of which five major effects are discussed in this review: the impact on immune responses, induction of developmental arrest, increases in lipid levels, apoptosis and nutrient releases. The value of deciphering this venom is also discussed.

## 1. Introduction

**Figure 1:**
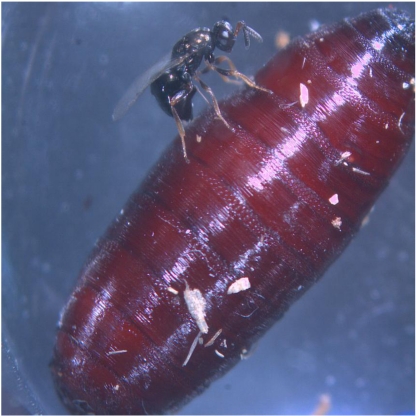
Female *Nasonia vitripennis* injecting venom into a pupa of the blowfly *Calliphora vomitoria*. [Picture remains copyright of Prof. Dirk C. de Graaf].

Parasitic wasps are important natural enemies of a vast array of insects that can achieve pest status in agricultural systems. Several species are important biological control agents used in integrated pest management programs relying on augmentative and/or inoculative release strategies. These parasitoids also produce an incredible and unutilized pharmacopoeia of venoms that may serve as leads for developing new classes of synthetic chemical insecticides. One species of particular interest is the fly ectoparasitoid *Nasonia vitripennis*, the only parasitic wasp to have its entire genome sequenced. *Nasonia vitripennis* feeds and lays eggs on cyclorrhaphous Diptera, showing preference for large flesh fly pupae (Figure 1). 

Adult females always inject venom prior to oviposition, and the envenomated fly never survives the attack. The venom is highly potent to a wide range of agriculturally important filth flies [1]. The wasp is also of medical importance, since it can be used in biological control of the common house fly, *Musca* *domestica*, a major vector of human disease. Furthermore, its venom is very toxic to multiple developmental stages of several mosquitoes that are vectors of such diseases as malaria, encephalitis, yellow fever and West Nile fever [2].

Envenomation of the host results in systematic alterations of the fly’s physiology. The initial research on the venom system of *N. vitripennis* revealed the protein-producing, insecticidal nature of the acid gland and the venom reservoir, and the non-toxic properties of the alkaline gland [3,4]. From then on, the function of *Nasonia* venom gained interest and a lot of research has been done by David B. Rivers and colleagues, mostly by performing bio-assays using fluids collected from the female reproductive system. Results from those assays suggested that the venom operated by nonparalytic means to induce an arrested or delayed development in envenomated hosts [5]. Venom also altered fly lipid metabolism, leading to lipid accumulation in the host fat body [6]. *In vitro* analysis of *Nasonia* venom showed that it could change plasma membrane permeability in different cell lines [7]. Subsequently, it was demonstrated that this causes an increase in Na^+^ influx, probably resulting in venom-induced cell death [8]. When fly hemocytes were examined after an incubation with *Nasonia* venom, suppression of host cellular immune response was suggested to be essential for successful feeding by the developing wasp larvae [9]. 

Several studies aimed at identifying the venom components involved in these processes. HPLC fractionation of venom from *N. vitripennis* isolated two low molecular weight proteins, apamin and histamine [10]. Enzymes responsible for phenoloxidase/L-DOPA oxidizing activity and a protein resembling calreticulin were discovered thereafter [11,12] respectively. The recent availability of the *N. vitripennis* genome sequences, has yielded an extraordinary amount of identified constituents [13]. Seventy-nine venom proteins were identified, of which 23 showed no similarity to any known protein. Now the enormous challenge remains to unravel the specific functions these proteins perform in influencing host physiology. In the present paper, possible biological functions are predicted based on homolog studies. Five major effects of *Nasonia* parasitization on the host are discussed: the impact on immune responses, induction of developmental arrest, increases in lipid levels, apoptosis and nutrient release. Possible interactions of the discovered venom proteins with host pathways are suggested. Although this exercise is mainly speculative, it seems to us an interesting starting point for further research.

## 2. Venom and Its Impact on Immune System

Female parasitoids face the challenge of conditioning the host to maximize progeny production, while avoiding ‘excessive’ manipulation that leads to rapid deterioration of the host. For example, induction of development arrest and stimulation of nutrient production/release are commonly triggered through the action of wasp venoms and other factors of maternal origin (*i.e.*, endosymbiotic viruses, VLPs) in an attempt to create an optimal environment for wasp offspring feeding and development [14,15]. However, if maternal agents like venom evoke a total suppression of host physiology leading to a completely immuno-compromised host, then unregulated microbial attack may occur. The result is the unfavorable host environment in which the parasitoid’s progeny compete directly with microorganisms for host nutrients, and in turn, face direct invasion by the microbes. Such scenarios may occur with both endo- and ectoparasitic wasps. It is thus not surprising that venom proteins with potential immunosuppressive and stimulatory properties were identified in venom from *N*. *vitripennis*. 

### 2.1. Immune suppression

Immunity plays a major role in physiological interactions between hosts and their parasitoids. Hosts will react to the invasion of foreign agents by producing antimicrobial peptides and reactive oxygen species by contact epithelia, fat body and hemocytes and more directly by phagocytosis, encapsulation and nodule formation in which specialized hemocytes interplay. The female wasp needs to avoid or evade this host immune response by introducing a venomous mixture, often together with virus-like particles and/or polydnaviruses [16], at the time of oviposition to subdue the host. *Nasonia vitripennis* injects a toxic venom into its hosts of which some of the recently discovered compounds possibly contribute to host immune suppression. Some components in this venom could have a potential function in targeting two major host defense cascades: the phenoloxidase cascade and the coagulation cascade. In addition, we will name a number of venom proteins from which the immune suppressive function is uncertain and/or purely speculative.

#### 2.1.1. Phenoloxidase cascade

**Figure 2:**
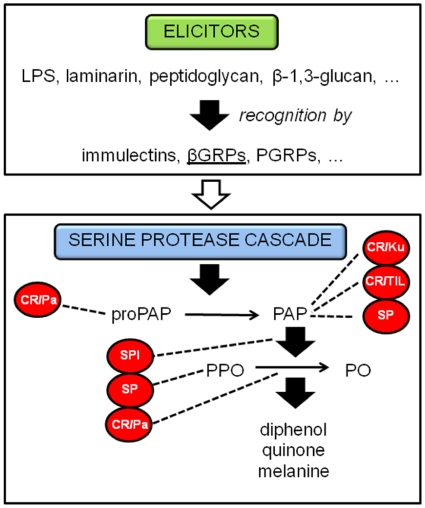
Phenoloxidase cascade in insects with its possible inhibitory and stimulatory proteins of which homologues were found in *N. vitripennis* venom. Abbreviations: LPS = lipopolysacharide, βGRPs = β-1,3-glucan recognition proteins, PGRPs = peptidoglycan recognition proteins, CR/Pa = cystein-rich/Pacifastin venom protein, SPI = serine protease inhibitor, SP = serine protease, CR/Ku = cystein-rich/Kunitz venom protein, CR/TIL = cystein-rich/trypsin inhibitor-like venom protein, (pro)PAP = (pro)phenoloxidase-activating protease, (P)PO = (pro)phenoloxidase. Words in red indicate inhibitory proteins and the dotted lines indicate their targets in the pathway; underlined words indicate stimulatory proteins.

A major innate defense system in invertebrates is the melanization of pathogens and damaged tissues. This important process is controlled by phenoloxidase (PO), a multicopper oxidase, which results in the deposition of melanin around the damaged tissue or intruding object. This physical shield around the intruder prevents or retards its growth and, perhaps even more importantly during melanin formation, will produce highly reactive and toxic quinine intermediates. These quinones are also involved in the production of cytotoxic molecules, such as superoxides and hydroxyl radicals, which could aid in the killing of invading microorganisms. Following oviposition by *Nasonia*, there is no evidence of hemolymph clotting at the wound site. However, melanization occurs within a matter of minutes in tissues penetrated by the ovipositor [9], and remains localized unless the fly is a non-permissive host, in which case melanization and tissue necrosis may spread rapidly from the site of venom injection [17]. David B. Rivers and colleagues suggested that the female wasp must inject factors into the fly host that retard or inhibit clotting and/or wound healing processes [9]. Reduction of PO activity is a well-known strategy of parasitoids, although so far reported almost exclusively in braconid and ichneumonid species [18,19]. The proteomic study on the venom composition of *N. vitripennis* done by Dirk C. de Graaf and colleagues [20], rendered three groups of proteins with possible inhibitory function on the phenoloxidase pathway, contributing to the prevention of encapsulation of endoparasitoid eggs. Figure 2 shows a presentation of potential targets of these protein groups on the PO cascade.

Three proteins found by the proteomic study belong to the group of serine protease inhibitors, or often referred to in the literature as *serpins*. In insects, several biological functions have been proposed for serpins [21]. The first one reported to block prophenoloxidase activation was serpin-1J [22] in the hemolymph of *Manduca sexta*. There is also evidence of serine protease inhibitor activity in the ovarian fluids of some endoparasitoid wasps [23,24]. These inhibitors have been shown to reduce host PO activity, although their exact targets in the cascade are not known. Two serpins in *M*. *sexta*, serpin-3 and serpin-6 [25,26] respectively, inhibit individual ProPO-activating proteinases (PAPs). PAPs are preferential targets of serpins to block the PO cascade. 

Protease inhibitors of the cysteine-rich venom protein subgroup are also involved in the inhibition of proPO Activating Proteases. LMPI-1 and LMPI-2 from *Locusta migratoria* are members from the Pacifastin inhibitors and show a regulatory role in the prophenoloxidase cascade by inhibiting PAP in crayfish hemolymph [27]. Cvp1, a cysteine-rich protein from the endoparasitoid *Pimpla hypochondriaca*, was proposed to be a phenoloxidase inhibitor [28]. It was suggested that this protein stabilizes or inhibits venom PO whilst it is stored in the venom sac. Another parasitoid related PO inhibitor has recently been presented, Egf1.0 [29]. This polydnavirus protein was found in a bracovirus carried by the wasp *Microplitis demolitor* and targets PAP during the host melanization response. Recently, 5 cysteine-rich proteins were discovered in venom from *N. vitripennis* [20]. 

The capsules trapping invading microorganisms and parasites require activation of a series of serine proteinases. One of these serine proteinases is PAP, which also exists as an inactive zymogen that becomes fully activated when serine protease homologs (SPHs) serve as its cofactor/anchor. These proteins are present in the plasma of several insects [30] and are non-catalytic serine proteases due to mutations in one or more catalytic residues. Interestingly, parasitoid venoms use SPHs as an antagonist molecule competing with host SPHs for binding sites of immunolectins and proPO, instead of activating the complex. *Cotesia rubecula* injects a venom protein, Vn50, into its host, *Pieris rapae* [31]. It could down-regulate proPO activation mediated by *M. sexta* PAP-1, SPH-1 and SPH-2, so reducing the proteolysis of proPO instead of inhibiting active PO or PAP-1. Proteomic analyses of venom from *N. vitripennis* revealed several serine proteases, of which some may have possible functions in down-regulating the PO cascade.

In *N. vitripennis* venom, two laccase proteins were found. Laccase belongs to the group of proteins known as multicopper oxidases and is hypothesized to play an important role in insect cuticle sclerotization. The venom protein lac1, found in *P. hypochondriaca*, showed L-DOPA oxidizing activity and has similar PO activity as the enzyme phenoloxidase [32]. It was suggested that venom PO derived products, if produced in sufficient quantity, could bind to hemocytes and disrupt their function, thus contributing to host immune suppression. But with *N. vitripennis*, no melanization occurred in cell culture media nor does host hemolymph melanize following envenomation [9]. Interestingly, venom phenoloxidase activity in *Nasonia* was found to be critical in the intoxication pathway leading to cell death (see below). 

#### 2.1.2. Coagulation cascade

**Figure 3:**
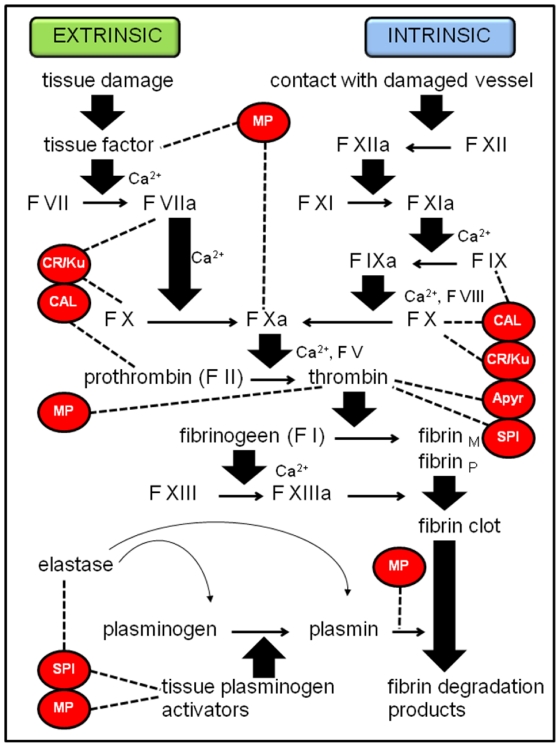
Coagulation cascade in vertebrates with its possible inhibitory proteins of which homologues were found in *N. vitripennis* venom. Abbreviations: F = factor, fibrin_M_ = fibrin monomer, fibrin_P_ = fibrin polymer, MP = metalloprotease, CR/Ku = cystein-rich/Kunitz venom protein, CAL = calreticulin, SPI = serine protease inhibitor, Apyr = apyrase. Words in red indicate inhibitory proteins and the dotted lines indicate their target molecules in the pathway.

The clotting reaction of insect hemolymph, which is based on a combination of soluble and cell-derived factors, is a part of insect immunity. *Nasonia vitripennis* venom contains 5 groups of proteins that can present a possible inhibitory function on the coagulation cascade of the host. After the female wasp has injected venom into the host and has laid her eggs, she often starts making a feeding tube (dependent on whether the female has previously oviposited before [33]), connecting the interior of the pupa with the exterior of the puparium [34]. Inhibition of coagulation is of crucial importance in this process, since the wasp feeds on the host fluid drawn up through the feeding tube. In addition to this, the young parasitoid larva also feeds on the host, grabbing and puncturing the host’s integument with its mandibles and taking in the host’s body fluids. Richards and Edwards [35] argued that the ectoparasitoid *Eulophus pennicornis* relies entirely on salivary secretions from larvae to knock out host defenses. A similar scenario was reported for the ectoparasitoid *Euplectrus plathypenae* [36]. Larval secretions are known to inhibit clotting but they can also contribute to the action of maternal venom. The acquisition of unclotted body fluids is probably (maybe partly) established by injecting the venom proteins suggested below. Figure 3 shows the speculative targets of these venom proteins on the vertebrate coagulation cascade. Although many arthropod clotting factors are not orthologues of blood clotting factors, they show novel architectures assembled from domains that are also found in their vertebrate counterparts. Therefore, the potential targets of the venom proteins can represent a combination of protein domains that are assembled in a different way instead of being real orthologues of proteins in the vertebrate coagulation system [37]. 

The first protein that could have an inhibitory function on the coagulation cascade of the host is a metalloproteinase. Some snake venoms and tick saliva have been found to contain metalloproteases, which confer antihemostatic and/or antifibrinogen specific activities [38,39]. A reprolysin-type zinc metalloprotease was found in the endoparasitoid wasp *P. hypochondriaca*, and it was suggested that it could act as an effecter of host immune suppression [40]. Just as in snakes, these proteinases are involved in the prevention of ‘blood’ clotting following envenomation. The purpose of clotting inhibition in snakes by metalloproteases is mainly for a more effective spread of its venom and gaining a better digestible prey. A similar function can be assigned to parasitoid venom (see above). Although with *P. hypochondriaca*, the prevention of clotting serves to inhibit accumulation of hemocytes around the parasitoid eggs. 

Although we are still far from molecular understanding of coagulation in insects, our knowledge about the defense system in horseshoe crab hemocytes has advanced greatly the last few decades [41]. In 1995, LICI, *l*imulus *i*ntracellular *c*oagulation *i*nhibitor has been discovered [42]. This serpin specifically inhibits limulus lipopolysaccharide-sensitive serine protease factor C, which takes part in the coagulation cascade in horseshoe crabs. Some blood-sucking insects are known to have serpins in their saliva that interfere with hemostasis, for example the tick *Ixodes ricinus* and the vector of Chagas’ disease *Rhodnius prolixus* [43,44]. The ovarian calyx fluid of the endoparasitoid *Venturia canescens* has the potential to alter host hemocyte spreading due to the presence of a putative serine protease inhibitor [23]. It was suggested that this serpin interacts with a host enzyme that has a similar function as thrombin, thereby inhibiting spreading of the hemocytes. Analyses of *Nasonia* venom by proteomics revealed two Kazal-type serine protease inhibitor-like proteins and one small serine proteinase inhibitor-like venom protein. 

The saliva of ticks also often contain yet another anticoagulant peptide that belongs to the Kunitz-type protease inhibitor family. Tick anticoagulant peptide (TAP), ixolaris and penthalaris were isolated from the saliva of two different tick species [45,46,47]. They bind to and inhibit coagulation factor X/Xa and exhibit factor Xa-dependent factor VIIa/tissue factor inhibitory activity. The venom of *N. vitripennis* contains 5 cysteine-rich venom proteins, of which one also contains the Kunitz domain (Figure 3).

Calreticulin is a highly conserved and ubiquitous protein that is a calcium storage depot in the endoplasmic reticulum and participates in calcium signaling. The highly acidic C domain binds the clotting factors IX, X and prothrombin [48]. Kuwabara S. and colleagues suggested that calreticulin, when infused into the intravascular space, binds to endothelial cells, blocks platelet adhesion and activation, and thereby prevents thrombus formation in canine coronary arteries [49]. A calreticulin-like protein was detected in *Nasonia* venom almost simultaneously by immunoblotting [12] and genome mining [20]. 

Mammalian platelets can be activated by many factors, for example by ADP that recruits and activates more platelets, inducing platelet aggregation. Apyrases hydrolyse ADP and therefore inhibit collagen- and thrombin-induced platelet reactivity, confirming the major role these proteins play in the prevention of thrombus formation. Apyrase activity is also present in the saliva of haematophagous arthropods, like *Triatoma infestans* [50] where it is related to blood feeding. It was remarkable to find a similar apyrase in *Nasonia* venom (Figure 3). 

#### 2.1.3. Other potential immune suppressing compounds

Both targeted cascades represent direct immune suppression towards the intruders, the parasitoid eggs. However, since suppression of these cascades typically occurs with endoparasitic species to protect the eggs/larvae, the exact significance to an ectoparasitoid like *N. vitripennis* still needs to be determined. Furthermore, some venom components are assumed to target the overall defense system of the host instead of blocking direct encapsulation or clotting around the invading parasitoids. The following venom components show strong similarities to proteins that weaken the host by preventing initiation of defense mechanisms.

Although metalloproteases have been speculated (see above) to target the PO and the coagulation cascade, they can also function in other anti-immune mechanisms. PrtA, a serralysin-type metalloprotease, was found in *Photorhabdus luminescens* (Enterobacteriaceae) involved in its host-pathogen interaction with *M. sexta* [51]. This virulence factor specifically cleaves immune proteins in the hemolymph of the host insect, rendering the host unable to establish the proper immune responses against possible threats. 

The braconid wasp *Toxoneuron nigriceps* is an endoparasitoid that controls its host’s development by polydnavirusses, secreted together with its venom, and by teratocytes. The latter are large cells present in the hemolymph of the host, that typically originate from the anterior and posterior serosal cells that surround the parasitoid embryo during its development. After *T. nigriceps* venom injection, teratocytes produce a putative chitinase that appears just before the egression of the parasitoid larva from the host. This chitinase was suggested to be involved in the facilitation of the parasitoid larva egression by aiding the digestion of the host cuticle, since the parasitoid larva lacks an elaborate mandibular apparatus [52]. *Nasonia vitripennis* venom also contains a chitinase, but since this wasp is an ectoparasitoid and therefore no larval egression is necessary, another function should be attributed to this protein. Often, chitinases are involved in breaking the physical barriers and gaining access to the host to initiate an infection process. This occurs with the malarial parasite *Plasmodium*, in which the protozoan’s chitinase digests the peritrophic membrane (which consists of chitin) of the mosquito [53]. This peritrophic membrane is produced by the gut epithelium and surrounds the blood meal, acting as a barrier for invasion of ingested microorganisms. By breaking down the barrier, the parasite can gain access to the host. 

Fungal acid phosphatases may target the immune system of the desert locust *Schistocerca gregaria* [54]. In this example, dephosphorylation of immune proteins that have been activated by phosphorylation may retard or disable host defenses. Acid phosphatases are also present in the venom of *N. vitripennis*.

Calreticulin was already hypothesized to have a potential inhibitory function on the encapsulation of the developing parasitoid through the PO cascade. But in the endoparasitoid *C. rubecula*, calreticulin also showed an inhibition of encapsulation, but through a different mechanism [55]. In this case, the venom calreticulin might function as an antagonist, competing for binding sites with the host calreticulin from the host hemocytes, which mediates early-encapsulation reactions. In addition, in humans, an interaction between calreticulin and complement component C1q is well documented [56]. Because calreticulin blocks the C1q-immunoglobulin interaction, it was hypothesized to be a potent inhibitor of C1q-dependent hemolytic activity [57]. The recent discovery of C1q and calreticulin in the *N. vitripennis* venom gland transcriptome [58] might point to a possible interaction. Further investigation is necessary to unravel the true function of calreticulin in *Nasonia* venom.

### 2.2. Immune stimulation

Venom from *N. vitripennis* is known for its suppressive potential towards the immune system of its hosts. But if host immunity is shut down completely, the host would become highly susceptible to external microbial threats with no means of defense. Such a scenario is potentially deleterious to the parasitoid’s developing progeny, which conceivably would be forced to compete with microorganisms for host nutrients, and could either directly infect/attack the wasp offspring and/or contaminate their nutritional source. Thus, one might expect that the parasitoid’s venom suppresses host immunity only selectively, for instance by interfering with host melanization and coagulation responses, yet allowing or even stimulating certain antimicrobial defenses. Here we will list a number of *Nasonia* venom proteins that could attribute to immune stimulation. Some reports are more speculative than others. 

A chitin binding-like protein is one of the components present in the venom of *N. vitripennis*. Chitin is a component of the cell wall of fungi, but is also the major structural component of arthropod exoskeletons. In the horseshoe crab *Tachypleus tridentatus*, tachycitin, a small granular component in its hemocytes was found to have antimicrobial and chitin-binding activities [59]. Kawabata and colleagues suggested that tachycitin is able to recognize chitin exposed at integumental lesions and to serve not only as an antibacterial molecule against invading microbes but also in wound healing, which may stimulate and accelerate biosynthesis of chitin at sites of injury. Besides the possible antibacterial function, chitin-binding proteins may also represent a structural component of the epicuticular lining of the venom gland to protect the secretory cells from the toxins they produce. This function was suggested by Nico Peiren and colleagues [60] for two endocuticular structural proteins with chitin binding activities in the venom gland of the honey bee worker. 

β-1,3-Glucan recognition proteins (βGRP) have strong specific affinity for β-1,3-glucan, a component of the fungal cell wall. In *Bombyx mori*, the synthesis of βGRP is induced by a bacterial or yeast challenge, although the gene is constitutively expressed in the hemocytes, fat body and epithelial cells [61]. Its interaction with β-1,3-glucan initiates the activation of the prophenoloxidase cascade. *Drosophila melanogaster* possesses at least three members of the Gram-negative bacteria-binding protein (DGNBP) family, one of which, DGNBP-1, has already been characterized [62]. DGNBP-1 was suggested to function as a pattern recognition receptor for lipopolysaccharides from Gram-negative bacteria and β-1,3-glucan from fungi and mediates innate immune signaling for the induction of antimicrobial peptide gene induction. A β-1,3-glucan recognition protein is also present in the venom of *N. vitripennis*.

Dipeptidyl peptidase IV (DPP IV) is part of the prolyl endopeptidase family within the class of the serine proteases. It is a membrane-anchored enzyme that sequentially cleaves dipeptides ending in proline or alanine. The human DPP IV (or CD26) is a multifunctional type-II membrane bound glycoprotein with at least 5 different functions: (1) serine protease, (2) receptor, (3) co-stimulatory protein, (4) adhesion molecule for collagen and fibronectin and (5) is involved in apoptosis [63]. DPPIV/CD26 plays a major role in human immune responses by activating substrates involved in inflammatory processes. DPP IV is also present in venom from snakes, scorpions, spiders, wasps and honeybees. In honeybee venom, it is called a “venom trace element,” because it occurs in extremely low quantities and has only a local function in the venom duct or reservoir [64]. It activates the toxin mellitin in the venom duct and most likely has no function once it is injected in the victim. Since DPP IV is also present in venom from *N. vitripennis*, it could possibly function as a stimulator of venom related processes, including immunity. 

Insect angiotensin I-converting enzyme (ACE) is a soluble single-domain peptidyl-dipeptidase of which the role in insects remains to be elucidated. In *L. migratoria*, it was suggested to be involved in peptide processing, more specifically by hydrolysis of lysyl-arginine and arginyl-arginine from the C-terminus of a prohormone peptide [65]. In the venom from the endoparasitic wasp *P. hypochondriaca*, ACE-like enzyme activity was detected [66]. This venom protein was found to be one of the processing enzymes involved in the synthesis of the antibacterial peptide, peptide B, from the C-terminal region of pro-enkephalin. It was stated that antibacterial factors present in venom may help guard against pathogens transferred to the host *via* the orifice created by oviposition. ACE was also found in the venom of *N*. *vitripennis*. 

Human neutrophils contain serine proteases and a serine protease homologue with antimicrobial activity. These proteins are called serprocidins and are located in azurophil granules, which are specialized lysosomes of the cells. In horseshoe crab hemocytes, a serine protease homologue, limulus factor D, belongs to this family and is co-released with other defense molecules stored in granules, in response to external stimulation of LPS [67]. Serine proteases have a conserved role in the activation of insect immune reactions, for example in *Drosophila*, of which the Toll pathway is well studied. The Toll receptor is activated by Spatzle, which is processed by complex cascades of serine proteases. It has been demonstrated that the serine protease ModSP integrates signals from recognition molecules and connects them to the pathway upstream of the Toll receptor [68]. Serine proteases and a serine protease homologue have been found in venom from *N. vitripennis* and a few possible functions of this important group of proteases have been proposed above.

A series of protease inhibitors was found in venom from *N. vitripennis*, of which two are Kazal-type inhibitors and one is a Kunitz-type inhibitor. Interestingly, both types of inhibitors have been found in the silk of the wax moth, *Galleria mellonella* [69]. These *s*ilk *p*roteinase inhibitors, SP1 and SP2, are involved in the antimicrobial defense by inhibiting bacterial subtilisin and fungal proteinase K. The cocoon silk protects the larval instars from microbes, fungi and mites, when it has spun its cocoon. Activities of these inhibitors in the wasp’s venom on bacterial and fungal proteinases remain to be elucidated. 

## 3. Venom and Developmental Arrest

Wasp parasitoids use a variety of methods to modulate their insect hosts in order to create an environment that will support and promote their progenies development, usually to the detriment of the host insect. Parasitized insects typically undergo developmental arrest and die sometime after the parasitoid has become independent of its host. Teratocytes from the endoparasitoid wasp *Microplitis croceipes* inhibit growth, alter development and affect related physiological parameters of *Heliothis virescens* larvae [70]. Furthermore, polydna virions coinjected with venom can induce a variety of physiological changes in development and immunity through expression of their genome genes [71]. David B. Rivers and colleagues [72] suggested that the venom of *N. vitripennis* is nonparalytic and development of envenomated hosts is either arrested or delayed. It has been demonstrated that host developmental arrest by this venom is not due to ecdysteroid deficiency [5]. The recent identification of functional proteins in venom from *N. vitripennis* should lead to further investigation on the exact venom proteins responsible for induction of host developmental arrest, as well as determination of the pathways manipulated by the venom. A few estimates are made below.

In this context, it was most interesting to find a protein with great resemblance to Imp-L2, which refers to *i*maginal *m*orphogenesis *p*rotein-*l*ate 2 in *Drosophila* [73]. Imp-L2 is a member of the immunoglobulin superfamily with an essential developmental role during embryogenesis. It is induced by the molting hormone 20-hydroxyecdysone and is suggested to be involved in regulating the availability or activity of *Drosophila* insulin-like peptide [74]. It has recently been characterized as a putative homologue of vertebrate IGF (insulin and insulin-like growth factor)-binding protein 7 that binds to *Drosophila* insulin-like peptide 2 (Dilp2). Imp-L2 is identified as a functioning insulin-binding protein that inhibits growth non-autonomously and would therefore be a possible arrestment factor in *N. vitripennis*. 

Metalloproteases can function as anti-immune proteins as presented above, but they also have roles in the control of development. EpMP3 is a reprolysin-like metalloproteinase and is a functional component of the venom from the parasitic wasp *Eulophus pennicornis* [75]. After injection of recombinant EpMP3 into its host, fifth instar *Lacanobia oleracea* larvae, its development is retarded with a reduction of growth and a failed moulting process. Since a metalloprotease is present in venom produced by *N. vitripennis*, it would be interesting to explore its possible contribution in immune suppression and/or on developmental arrest of the host.

During the moulting period of an insect, an increase in lysosomal activity in tissues such as the fat body occurs. Lysosomes in the southern armyworm (*Prodenia eridania* Cramer) are known to produce arylsulphatase, which might have an important physiological role in regulating the concentrations of insect moulting hormones [76]. Together with sulphotransferases, arylsulphatase could deactivate, store and reactivate insect moulting hormones through a process of enzymic sulphoconjugation and sulphohydrolysis. The arylsulphatase identified in *N. vitripennis* might therefore interfere in the development of the host.

The mode of action of calreticulin has already been discussed in immune suppression of the host. But the calreticulin-like venom protein from *N. vitripennis* also appears to be a critical agent in developmental arrest [12]. When venom calreticulin is bound with antibodies, most injected flies could complete pharate adult development to eclosion. When exogenous calreticulin was added, venom’s ability to retard fly development was restored. Rivers D.B. and Brogan A. speculated that venom calreticulin appears to mobilize intracellular calcium in susceptible cells. However, this protein cannot cross the plasma membrane on its own, and may rely on venom PO to function as a ‘carrier’ or a molecule that stimulates changes in membrane structure to facilitate entry of calreticulin into target tissues. Much more work is needed to elucidate the role of both types of venom proteins in developmental arrest. 

## 4. Venom and Stimulation of Increments of Lipid Levels

Parasitism of the flesh fly, *Sarcophaga bullata* by *N. vitripennis* causes a lipid accumulation in the fat body [6]. A proposed pathway for this lipid elevation was presented by Rivers and colleagues [8]. Injection of wasp venom into the host involves a change in plasma membrane permeability followed by an influx of Na^+^. This could trigger PLC activation, resulting in IP_3_ formation and subsequent Ca^2+^ release from mitochondria. In this scenario calcium release subsequently activates PLA_2_, which in turn stimulates fatty acid synthesis in host fat body. This way of elevating host lipids may be a strategy employed by the female wasp to maximize the fly as a resource for progeny production. The exact component in the venom that induces the change in plasma membrane permeability still needs to be found.

In insects, fat body cells perform endocytic uptake of circulating high density lipophorin (HDLp) which carry diacylglycerol (DAG) as their major neutral lipid cargo. An insect homolog of the vertebrate very low density lipoprotein receptor was found to mediate this endocytosis of lipophorins [77]. The low-density lipoprotein receptor-like venom protein found in venom from *N. vitripennis* could possibly be responsible for the internalization of HDLp in the fat body.

## 5. Venom and Apoptosis

Venom from *N. vitripennis* induces cellular injury and culminates in oncotic death. The cells also undergo apoptosis, displaying extensive membrane blebbing and condensation of nuclear material. Formation of blebs usually involves the disruption of cytoskeletal-membrane interactions and depends on rearrangements of intracellular Ca^2+^ levels [78]. Venom elicits a rapid loss of mitochondrial membrane potential, followed by unregulated Ca^2+^ efflux into the cytosol. This calcium mobilization could stimulate cAMP formation and subsequently promotes calcium release, leading to apoptosis of the cell. 

The first two candidates in venom to trigger apoptotic pathways in the host would be calreticulin and laccase. These venom proteins appear to be critical factors in the intoxication pathway to induce cell death. When BTI-TN-5B1-4 cells were pre-treated with PTU (phenylthiourea, a potent inhibitor of venom phenoloxidase) and anti-calreticulin polyclonal antibodies, cytotoxic action of venom and increases in intracellular calcium were suppressed [12]. Laccase has phenoloxidase activity, which could evoke disruption of plasma membrane integrity in susceptible cells, blebbing, rounding and swelling [11]. Together with calreticulin, they could be involved in venom-mediated mobilization of intracellular calcium that ultimately leads to cell death.

Apoptosis also involves nucleosomal fragmentation of DNA, performed by nucleases. Endonuclease G is a mitochondrion-specific nuclease that translocates to the nucleus during apoptosis. It cleaves chromatin DNA into nucleosomal fragments independently of caspases [79]. *N. vitripennis* venom contains an endonuclease-like venom protein.

γ-Glutamyl transpeptidase (γ-GT) is an enzyme that catalyzes the transpeptidation reaction in which a γ–glutamyl moiety is transferred from γ–glutamyl compounds, such as glutathione and glutathione-conjugated compounds, to amino acids. The γ–glutamyl cycle is a highly balanced process in which γ–GT plays a central role in protecting the cells from oxidative stress by indirectly supporting the intracellular glutathione synthesis. Any disruption of this delicate glutathione balance may determine significant changes in cell behaviour. In humans, *Helicobacter pylori* infection of gastric epithelial cells induces apoptosis. The purified protein from *H. pylori* responsible for this induction of apoptosis appears to be γ-GT [80]. In host-parasite interactions, γ-GT is also able to induce apoptosis. Parasitism by the endophagous braconid *Aphidius ervi* causes castration of its host *Acyrthosiphon pisum*. γ-GT triggers apoptosis of the cells in the germaria and ovariole sheath of the host by changing the glutathione metabolism and causing a consequent oxidative stress [81]. Venom produced by *N. vitripennis* also contains a γ-glutamyl transpeptidase-like venom protein and this venom protein is a likely candidate involved in the apoptotic pathway(s) utilized in envenomated hosts. 

## 6. Venom and Nutritional Functions

Parasitoids regulate host physiology in an attempt to maximize the mother’s fecundity and to provide optimal conditions for offspring to feed and develop. Therefore, the nutritional and physiological environment of the host is manipulated by substances injected by the female wasp or other related factors. Teratocytes of endoparasitoids, for example, have a secretory and nutritive function to ensure a nutritional milieu for the offspring, while venom of some ectoparasitoids changes the host metabolism to provide nutrients. Venom from *N. vitripennis* contains enzymes presumed to be involved in assuring the optimal nutriment for its offspring. 

Trehalose is the main reserve sugar in the hemolymph of flying insects. To utilize hemolymph trehalose, insect tissues contain trehalases that catalyze the hydrolysis of one mole of trehalose to two moles of glucose. Insects are believed to have two types of trehalases, a soluble form (tre-1) and membrane-bound (tre-2) [82]. The discovery of tre-1 as one of the venom proteins synthesized by *P. hypochondriaca*, suggests that the activity of this enzyme is to provide glucose for developing wasp larvae. This would indicate a digestive function for parasitoid venom [83]. Trehalase was also found in the venom from *N. vitripennis*.

Several serine proteases are present in venom from *N. vitripennis* and different potential functions have been proposed above. But a great number of serine proteases are involved in digestion, with trypsins as the most extensively studied group. In the hard tick *Haemaphysalis longicornis*, the HlSP gene (*H. longicornis* Serine Protease) seemed to have similar enzymatic activity with trypsin and was involved in blood meal digestion [84]. The larvae of the ectoparasitoid *Euplectrus separatae* release saliva containing a trypsin-like enzyme to digest the host tissues [85]. 

A lipase-like venom protein and a lipase similar to the one found in *P. hypochondriaca* were found in venom from *N. vitripennis*. Studies on the larvae of the endoparasitoid *Cotesia kariyai* showed that feeding on the host fat body occurred with the help of teratocytes [86]. Seven days after parasitization, the total amount of lipid from the fat body of the parasitized hosts decreased, while lipase activity of the parasitoid larvae increased. This supported the theory that lipases can digest lipid granules in the gut of the host and therefore provide nutrients to the parasitoid larvae.

Acid phosphatases catalyze the hydrolysis of phosphoric esters to release carbohydrates and inorganic phosphate. There are a few reports of the presence of this protein in venom. In the honeybee *Apis mellifera*, this enzyme may serve as a predigestion enzyme of prey before it was eaten or fed to the young [87]. In parasite-host interactions, it is proposed to be involved in providing nutrients from the host. The fungus *Metarhizium anisopliae* releases acid phosphatases into the haemolymph of its host insects for fungal growth [88]. Its presence in venom from *P. hypochondriaca* provided a source of carbohydrate from the host haemolymph for the developing parasitoid larvae [89]. Acid phosphatases were also found in *Nasonia* venom. 

## 7. “Unplaced” Venom Proteins

The study on the venom composition from *N. vitripennis* also revealed the presence of some unplaced proteins that have not been described yet in the context of insect venoms. Their biological function remains obscure and these are listed below. 

The General odorant binding protein-like venom protein (GOBP-like venom protein) belongs to the group of Odorant-binding proteins (OBP’s) and is involved in the interaction between odorants and the elements of the sensillar lymph [90]. Inosine-uridine preferring nucleoside hydrolase serves to salvage the host purine nucleosides by catalyzing the hydrolysis of purine and pyrimidine nucleosides into ribose and the associated base [91]. The two found aminotransferase-like venom proteins belong to the kynurenine aminotransferase subgroup, involved in the transamination of kynurenine to an α–keto acid. There has been one reported in *Aedes aegypti* [92], but so far, none have been reported in venomous secretions. Glucose dehydrogenase is an enzyme in the pentose phosphate pathway and belongs to the family of oxidoreductases. Antigen 5-like protein is, in contrast with the other mentioned mysterious venom proteins, a known compound in venoms from Hymenoptera [93]. However, the biological function of antigen 5-like protein still needs to be elucidated. The two antigen 5-like venom proteins in *N*. *vitripennis* are the first discovered in parasitoid wasp species. 

Last but not least, out of the 79 discovered venom proteins in *N. vitripennis*, 23 were found to have no homology with known proteins and even lack a known conserved domain architecture. Consequently, it is impossible to predict their biological function right now. 

## 8. Conclusions and Future Prospects

*Nasonia vitripennis* is an ectoparasitic wasp of fly pupae and pharate adults, meaning that hosts are non-mobile and do not feed. This type of host-parasite association initially led some investigators to conclude that venom was not necessary for parasitism [94], or if it was used, venom merely served to kill the host, thereby ‘fixing’ or ‘preserving’ the nutrient pool available for *N. vitripennis* [95]. Not only does this wasp have an elaborate venom system, it produces venom that is always injected prior to oviposition and which is necessary for the successful development of the parasitoid’s progeny. As discussed in this review, venom can elicit a diverse range of host responses, including suppression of host immune responses to changes in the fly nutritional status, all for the benefit of wasp larvae. The fly does not simply become a finite resource following envenomation, rather it undergoes a series of regulated, sequential alterations that are synchronized with key developmental events of feeding larvae. At the end of the parasitic association, the envenomated host dies, and there is evidence that both apoptotic and oncotic pathways are activated. By this point, however, the fly has outlasted its physiological value to the parasitoid’s larvae. 

This very impressive manipulation of the fly host appears to be choreographed entirely by venom. Such biological and physiological diversity in host responses has led to previous speculation that venom from *N. vitripennis* contains a wealth of compounds with unique chemistries and modes of actions. Genome mining and proteomic analyses of venom glands have confirmed these assertions. Seventy-nine proteins were identified in venom, with known functions being assigned to forty-six of the venom proteins. Most importantly, we now have identified a series of potential candidates in the venom that have obvious potential roles in venom-mediated immunosuppression, induction of developmental arrest, elevation of host lipid levels, changes in nutrient release, and termination of the fly’s life through apoptosis and oncosis. The next step is to confirm the functionality of the seventy-nine proteins.

Deciphering the venom cocktail of *N. vitripennis* has value at many levels. At the applied level, several of the identified venom proteins may have considerable potential in the development of biorational insecticides. Crude venom demonstrates toxicity toward pest insects from three different orders [39], yet displays selectivity for specific fly and mosquito species [2,39]. The latter aspect is incredibly important as there are relatively few naturally occurring toxins with dipteran specificity available for use in the construction of selective bioinsecticides. The most widely used are produced by bacteria such as *Bacllius* *thuringiensis* and *B. sphaericus*, but are limited because the toxins are only active against aquatic stages of dipteran pests [96,97]. Thus, the specificity of venom, and presumably individual venom proteins, from *N. vitripennis* offers promise of adding powerful new chemical weapons to the arsenal of compounds used to control medically and agriculturally important fly pests. 

At the basic level of research, several of the identified venom proteins may serve as important tools for examining the mechanisms used to manipulate and modify the host condition. For example, wasp venom appears to contain proteins specific for at least two enzymatic cascades typically associated with host immune responses. The modes of action of these proteins will shed light not only on functionality of the venom proteins, but also with regard to general aspects of insect body defenses. Similarly, venom proteins have the potential to be used in understanding insect lipid metabolism since one or more of these proteins trigger lipid uptake/accumulation and/or inhibit nutrient release in fly fat body. It is also conceivable that multiple venom proteins can be tools used to decode key events in fly development; particularly pathways involved in developmental arrestment, such as seasonal hibernation programs like diapause.

The possible function of several of the proteins discovered in venom from *N. vitripennis* may also open up new doors for exploring the evolution of parasitism within the parasitic Hymenoptera. The proposed function discussed in this review for specific venom proteins in immunosuppression cascades, developmental arrest, lipid modifications, and apoptosis overlap with gene products from many polydnaviruses and baculoviruses [10]. In several instances, there appear to be similarities in target sites (e.g., hemocytes) within hosts and nearly identical host responses, suggesting a possible common ancestry between wasp venoms (from both endo- and ecto-parasitoids) and polydnaviruses. An intriguing research area to explore is in the possibility that venom may merely be an evolutionary remnant of a past symbiotic relationship between wasps and viruses [98,10]. Comparisons of venom gene sequences of the newly discovered venom proteins from *N. vitripennis* with viral genes from endo- and ecto-parasitic wasps is one step toward uncovering their evolutionary relationships. Such information offers to provide great insight into the modes of action of wasp venoms and other insect-selective toxins through comparisons of homologous regions between viral, bacterial and venom gene products, and using *in vitro* and *in vivo* bioassays. 
